# Mobility Matters: A Pilot Study on Increasing Health Literacy Among Fall-Risk Elders

**DOI:** 10.1093/geroni/igaa057.747

**Published:** 2020-12-16

**Authors:** Mary Milidonis, Jane Keehan, Rebecca Deuley, Sara Formoso, Katherine Montgomery, Karen Kopera-Frye

**Affiliations:** 1 Cleveland State University, Cleveland, Ohio, United States; 2 Cleveland State University, Cleveland, United States; 3 New Mexico State University, Las Cruces, New Mexico, United States

## Abstract

Mobility is important to sustain for older adults to live independently. The purpose of this project was to evaluate teach back and ask me 3 interventions with a health education program that included Otago strength and balance exercise and a walking program The pilot program, Mobility Matters, was completed with 16 older adults (mean age =76, range 63-87, SD = 8.6), 69% African American, 94% female. Older adults with moderate fall risk were recruited from community centers and participated in a 3-month program where they were paired with physical therapy students for pre- and post-intervention assessment. Participants were randomly assigned to a health literacy intervention group (HLG) (n=9) and received teach back and ask me 3 intervention twice a month for three months. The control group (n=7) received the same program of balance exercises/ walking program and after 3 months was given the health literacy intervention. Groups were not significantly different on age, gender and REALM scores. Assessment measures included: timed up and go, 30 second chair rise, 4 stage step test, 6 minute walk test, and activity balance confidence scale (ABC). Paired t-test analysis revealed mean significant differences on the measures of four stage balance test (p =.008), six-minute walk test (p=.026) and approached significance on ABC (p=.054). No significant differences were found for the non-health literacy group on all measures. The results suggest that health literacy intervention may improve outcomes for health education interventions with balance and aerobic exercise.

